# Editorial Brevities

**Published:** 1904-03

**Authors:** 


### EDITORIAL BREVITIES.

THE New York Pharmacal Co. have issued a booklet called "Facetiæ Medicorum," containing the best things which have appeared in the "Doctor's Factotum." If you haven't a copy send and get one. They will take pleasure in sending it to you free.

---

THE following new medical journals have been launched since January 1:

*Southern Medicine and Surgery*, Chattanooga, Tenn. Dr. Raymond Wallace, Editor, Dr. B. F. Travis, Managing Editor, with list of collaborators.

*The Medical Recorder*, Shreveport, La. Dr. Oscar Dowling, Editor, Drs. Louis Abramson and R. H. T. Mann, Associate Editors.

*Southern Medicine*, Savannah, Ga. Dr. W. E. Fitch, Editor. A revival of the defunct *Georgia Journal of Medicine and Surgery.*

